# Effect of feed restriction on the environmental variability of birth weight in divergently selected lines of mice

**DOI:** 10.1186/s12711-019-0471-9

**Published:** 2019-06-13

**Authors:** Nora Formoso-Rafferty, Isabel Cervantes, Juan Pablo Sánchez, Juan Pablo Gutiérrez, Loys Bodin

**Affiliations:** 1 Departamento de Producción Animal, Facultad de Veterinaria, Universidad Complutense de Madrid, Madrid, Spain; 2 Animal Breeding and Genetics Program, Institute for Food and Agriculture Research and Technology, Caldes de Montbui, Barcelona Spain; 3 Génétique, Physiologie et Systèmes d’Elevage, INRA, Castanet-Tolosan, Toulouse, France

## Abstract

**Background:**

Selection of mice for decreased environmental variability of birth weight has achieved higher survivability and larger litter size as a correlated response to canalized selection, which suggests higher welfare and robustness, and animals that are more homogeneous. However, in these studies, animals were not exposed to an environmental challenge. To demonstrate the advantages of this mouse line with a low environmental variability of birth weight, animals from two divergent lines (high and low variability of birth weight) were subjected to feed restriction. The objective of this study was to use these divergent lines to compare their response in terms of robustness against an environmental challenge. At weaning, 120 females, i.e. four full-sib females from 10 random litters of three consecutive generations of selection, were chosen from these divergent lines. The total number of females was divided into four groups, which were subjected to a feeding regimen by imposing different levels of feed restriction (i.e. 75, 90 and 85% of full ad libitum feed across three generations, respectively) in different combinations during the growth and reproduction periods.

**Results:**

Animals from the “low” line were less sensitive to a change in feed level than those from the “high” line. Regarding reproduction, the “low” line performed better in terms of number of females having parturitions, number of parturitions, and litter size. Imposing a feed restriction on female mice during their growth period did not affect the birth weight of their pups. The “low” line was preferred because of its higher reproductive efficiency and survival under an environmental challenge.

**Conclusions:**

Selection for decreased environmental variability of birth weight produces animals that are less sensitive to environmental conditions, which can be interpreted as having greater robustness.

## Background

Animal production requires the effective use of resources, i.e. animals that perform better and that are more robust. Moreover, sustainability is related to robustness, and robustness is related to homogeneity, but evaluating robustness and homogeneity in an experimental population has rarely been carried out [1]. Mormede and Terenina [2] suggested that robustness is the combination of a high production potential with a low sensitivity to environmental changes or to a certain level of stress.

Since several decades, feed costs represent the major part of breeding costs and are one of the major concerns for breeders. Different feeding strategies, such as feeding to appetite (ad libitum) or feeding under restrictions, are common practices in commercial animal production [3]. Efficient use of feed resources for growing animals is one of the major factors that influence the economic sustainability of animal production [4].

Selection for homogeneity, in general, can affect feed efficiency and may benefit productivity and animal welfare, even when feed restrictions are applied. However, feed restriction can affect both animal performance and welfare. In rabbits, it has been demonstrated that an ad libitum diet maximizes performance [5] increases sexual receptivity, ovulation rate, blastocyst size, and implantation rate [6].

Previously a divergent selection experiment was carried out for 17 generations in two mouse lines to gain more insight into the effect of selection for homogeneity of birth weight (BW) [7]. The study of these two ad libitum fed lines by Formoso-Rafferty et al. [1] included the evaluation of production, reproduction, and animal welfare traits. They showed that feed efficiency was similar in both lines, but that fertility and welfare were higher in the homogeneous line, which suggested higher robustness.

Previously, some authors reported that homogeneity of body weight was correlated with important reproductive traits such as fertility or litter size in rabbits [8–10] and with robustness traits such as welfare [2] or survival in piglets and rabbits [11–13]. However, in these studies, the animals were not exposed to any environmental challenges, which did not allow direct assessment of the change in performance under new environmental conditions. Animals with a performance that remains unchanged under an environment challenge can be considered as being more robust. Hence, the objective of our study was to analyze the influence of feed restriction, which is considered an environmental challenge, in two mouse lines that were divergently selected for environmental variability of birth weight (BW).

## Methods

Data were recorded on two mouse lines that were divergently selected for environmental variability of BW. Details of the selection process are in Formoso-Rafferty et al. [14]. In the current paper, “low” and “high” will be used for the line with, respectively, a low and high environmental variability of BW. Briefly, this selection experiment demonstrated a divergent response for the residual variance of BW and that it was positively correlated with traits related to welfare and robustness.

Data for animals from three consecutive generations of the selection experiment (12, 13 and 14) were available. Hence, 40 females (four full-sib females from 10 random litters) per generation and line were chosen at weaning, divided into four treatment groups (1 full-sib per group) that were assigned different combinations of feed restriction during the growth and reproduction stages.

First, the 40 females per line were divided into two groups (20 females per group), with one group fed ad libitum and the other with a restricted diet during the growth period (GP). The effect of the feeding regime during the GP was noted as R_GP. Female body weight in g (W) was recorded weekly from weaning to 3–11 weeks of age. Starting at 11 weeks of age, both groups were fed ad libitum (recovery period) until the start of the reproduction period (RP).

To explore the effects of line and feeding regime (ad libitum or feed-restricted) on feed efficiency during the GP, the cumulated transformation index was computed for each of the 10 weeks after weaning as the ratio of cumulative feed intake (in g from weaning to the specific week) and weight gain (in g from weaning to the specific week). The last 2 weeks of this 10-week period were a recovery period during which all animals were fed ad libitum.

**Fig. 1:**
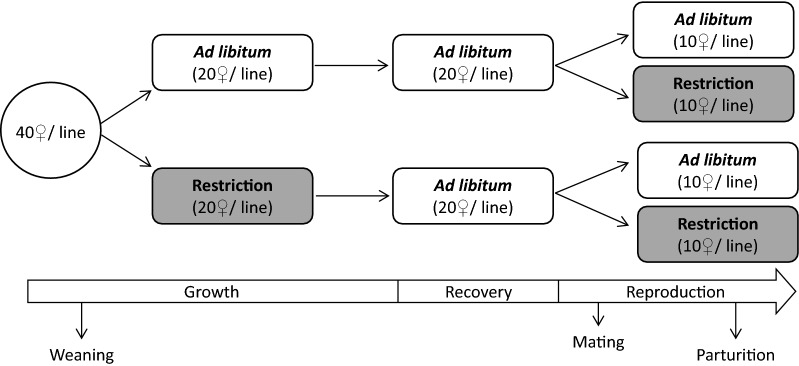
Design of the post-weaning experiment and distribution of the females in the corresponding experimental group according to their feeding regimen (ad libitum or restricted) during growth and reproduction periods

One week before mating, each GP feeding group was divided into two new groups (Fig. 1): one was fed ad libitum and the other with a restricted feed regimen during the RP. Thus, the resulting four groups had a combination of ad libitum and restricted feeding regimes during GP and RP (Fig. 1). The effect of the feeding regime during the RP was identified as R_RP.

During the RP, each female cohabitated with one male from the same line for two reproduction cycles to give the female the opportunity to become pregnant twice. Pregnant females were checked daily, litter size was recorded within 24 h after birth, and the new-borns were weighed and identified individually. The number of females with only one (P1) or two (P2) parturitions were also recorded. P1 was recorded on all 40 females that were available within line and generation, while P2 was recorded only on the females that had a P1. An overall measurement of fertility (%) was also recorded within line, generation, and feeding regime, as the percentage of available females that had a parturition. In generation 12, survival (%) was also measured as the percentage of females alive compared to the total number at the start of the experiment.

At the beginning of the experiment, in generation 12, and taking the current literature concerning feeding restriction on mice and other species [15–20] into account, we decided to apply a feeding restriction of 75% of the full ad libitum intake. The amount of feed provided to the restricted groups was 75% of the mean ad libitum consumption in the corresponding line at a particular physiological state (GP or RP) in the three previous generations [1]. Unexpectedly, 75% of ad libitum feeding resulted in increased mortality, particularly in the high line, which suggests that animals from this line were less robust. Because of this unexpected mortality, we decided to reduce the percentage of restriction in the subsequent generations, i.e. in the second generation (13), feeding restriction was modified to 90% of the ad libitum intake. In this particular case, some animals did not consume all the food provided, which strictly speaking means that the feeding regimen was not restricted. Thus, in the third generation (14), we applied a feeding restriction of 85%.

### Statistical model for female growth and fertility

The data used in this study included weekly weights for each female from weaning to 10 weeks of age, with an initial 240 records and a final count of 225 at the end of the experiment. The weights for each week were analyzed with a model that included the effects of R-GP (ad libitum or feed-restricted), line (low or high), generation (12, 13 or 14), the litter size in which the female was born (with 3 levels, i.e. from 4 to 9, 10 to 11 and 12 to 15 pups), and the first order interactions between line, generation, and R_GP. It would have been better to include also a genetic effect to separate the influence of selection from the environmental effect of the generation, but this was not possible because of the small number of animals in the dataset. The analysis was carried out with the Release 4.1 ASReml program [21]. Survival and fertility were compared based on single statistical Chi square tests using the FREQ procedure of the SAS software [22].

### Statistical model for birth weight of offspring

The dataset contained 1275 records for BW of the progeny of 116 females with at least one parturition, 158 litters, and 4900 pedigree records. The heteroscedastic model of SanCristobal-Gaudy et al. [23] was fitted to the BW data. This model assumes that the BW mean and its residual variance are both affected by genetic and environmental factors and can be written as:

$$ y = \mu + m + l + e^{{\left( {\eta + \nu /2} \right)}} \varepsilon , $$

where $\mu$ and $\eta$ represent the systematic effects that affect the mean and environmental variability of BW, respectively, $m$ and $\nu$ are random effects that account for the maternal genetic effects that affect the mean and variability of BW, respectively, and $l$, is a random litter effect with variance $\sigma_{l}^{2}$.

Vectors ${\mathbf{m}}$ and ${\mathbf{v}}$ of the genetic values were assumed to follow a joint distribution:

$$ \left( {\begin{array}{*{20}c} {\mathbf{m}} \\ {\mathbf{v}} \\ \end{array} } \right)\,\sim\,N\left[ {0,\left( {\begin{array}{*{20}c} {\sigma_{m}^{2} } & {\rho \sigma_{m} \sigma_{v} } \\ {\rho \sigma_{m} \sigma_{v} } & {\sigma_{v}^{2} } \\ \end{array} } \right) \otimes {\mathbf{A}}} \right], $$

where $\otimes$ denotes the Kronecker product and ${\mathbf{A}}$ is the additive genetic relationship matrix between animals.

Regarding the systematic effects, the model included the feeding regimen of the dam during the growth (R_GP) and reproduction (R_RP) periods (ad libitum or feed-restricted), line (low or high), generation (12, 13, or 14), the litter size (LS) that each dam was born in (LS: from 4 to 9, 10 to 11 and 12 to 15 pups; i.e. 3 levels), parturition number (P1 or P2), litter size of the progeny (LS_pup_: from 1 to 5, 6, from 7 to 9, 10, and from 11 to 13 pups; i.e. 5 levels), and the sex of the progeny (male, female, or unknown), along with the first order interactions between line, generation, and RP. A first analysis included all of these effects in the model but, in a second step, those that were not significant (*p* < 0.05) were removed. Only the results obtained from this second analysis are presented.

The pedigree information went up to the founding population of both lines and, thus, we could assess the genetic influence of line on BW by considering differences in the means of breeding value predictions for the animals involved in the experiment, which means that the systematic effect of line explains exclusively the environmental effects associated with line.

The heteroscedastic model was solved through a double hierarchical generalized linear model (DHGLM) developed by Felleki et al. [24] within the frame of ASReml (ASReml Release 4.1 software) [21]. Since this model assumes a residual variance for each level of combination of systematic effects [7], when required, the estimate of the residual variance for a given level of a given effect was obtained by averaging over the solutions of all other effects as:

$$ \hat{\sigma }_{{e_{sl} }}^{2} = e^{{i = 1,\sum\nolimits_{systematics}^{i \ne 1} {\left( {\sum\nolimits_{{j = 1,n_{s} }} {\frac{{\hat{b}_{ij} }}{{n_{s} }}} } \right)} }} + \hat{b}_{sl} , $$

where $\hat{b}_{ij}$ is the solution for a particular level $l$ of a systematic effect $s$, and $n_{s}$ is the number of levels of the systematic effect. In particular, the estimate of the residual variance for the combined effect of line and RP was obtained as:

$$ \hat{\sigma }_{{e_{sl} }}^{2} = e^{{\overline{sex} + \overline{{LS_{pup} }} + \overline{PN} + \overline{generation*RP} + \overline{GP*RP} + line*RP_{i} }} . $$

## Results

### Growth and fertility

Table 1 shows the differences in survival between lines and feed intake regimens at 10 weeks of age and at mating in generation 12 of the experiment, in which the feeding restriction was 75% of full ad libitum. At 10 weeks of age and at mating, there were significant differences in survival between feeding regimes (ad libitum or feed-restricted) in the high line, and between lines in the restricted feeding regimen.

**Table 1:** Female survival rate (%) during growth at 10 weeks of age and at mating in both the high and low environmental variability of BW lines, with ad libitum feeding or under feed restriction, in the first generation of the experiment

Line	Age	Ad libitum feed	Feed restriction
High	10 weeks	100^a^	65^bd^
	Mating	95^a^	60^bd^
Low	10 weeks	100	95^c^
	Mating	100	85^c^

a Versus b: significant differences within feeding regimen

c Versus d: significant differences within lines

Regarding W, Table 2 shows the significance level of the factors considered in the model at week 0 (at weaning), 1, 2, 3, 4, 5, 6, or 7 after weaning. There was no significant environmental influence of the line on any of the weights analyzed. The generation effect on W was highly significant (*p* < 0.001) at 2 weeks after weaning and later on. The effect of litter size on W was also highly significant (*p* < 0.001) throughout the experiment (weeks 0–7). As expected, the feeding regime (R_GP) showed a highly significant effect on W (*p* < 0.001) for all weeks after weaning (weeks 1–7).

**Table 2:** Significance of weekly female weights between lines, generations, feeding regimen during the growth period (R_GP), their interactions and litter size

	W_0_	W_1_	W_2_	W_3_	W_4_	W_5_	W_6_	W_7_
Line	ns	ns	ns	ns	ns	ns	ns	ns
Generation	ns	ns	***	***	***	***	***	***
R_GP	–	***	***	***	***	***	***	***
Line*generation	**	ns	ns	ns	**	ns	ns	ns
Generation*R_GP	–	**	***	***	***	***	***	***
Line*R_GP	–	ns	***	***	***	**	***	**
LS	***	***	**	***	***	***	***	***

*Ww* live weight (g) (in week w after weaning as a subindex), *LS* litter size, – effect not taken into account, *ns* not significant

**p < 0.01; ***p < 0.001

The interaction between line and generation was either not or weakly (*p* < 0.01) significant across the growth period, but the interaction between generation and R_GP was highly significant (*p* < 0.001 or *p* < 0.01), reflecting differences in the level of restriction applied in each generation. In fact, all first-order interactions for W were significant to some extent at some weeks. The significance of the interaction between line and R_GP on W was also high (*p* < 0.001 or *p* < 0.01) from weeks 2 to 7, which indicates that the impact of feed restriction differed between the two lines.

**Fig. 2:**
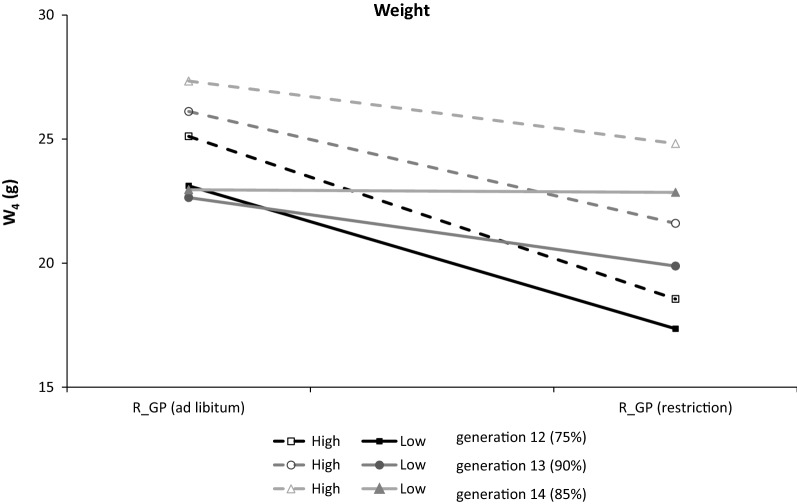
The least square mean estimates of female weight (in g) in the fourth week after weaning in the low line (continuous lines) and high line (discontinuous lines) depending on feeding regimen (ad libitum or restricted) in the three generations

Figure 2 shows the least square means for W at 4 weeks after weaning in both lines in the three generations analyzed. Week 4 was taken as a reference for the adult age of a mouse, but the pattern was similar for all weeks throughout the experiment. Irrespective of diet (ad libitum or restricted), females from the low line were slightly lighter than those from the high line and females that were fed ad libitum in the low line maintained a similar weight over all three generations, in contrast to those of the high line.

**Fig. 3:**
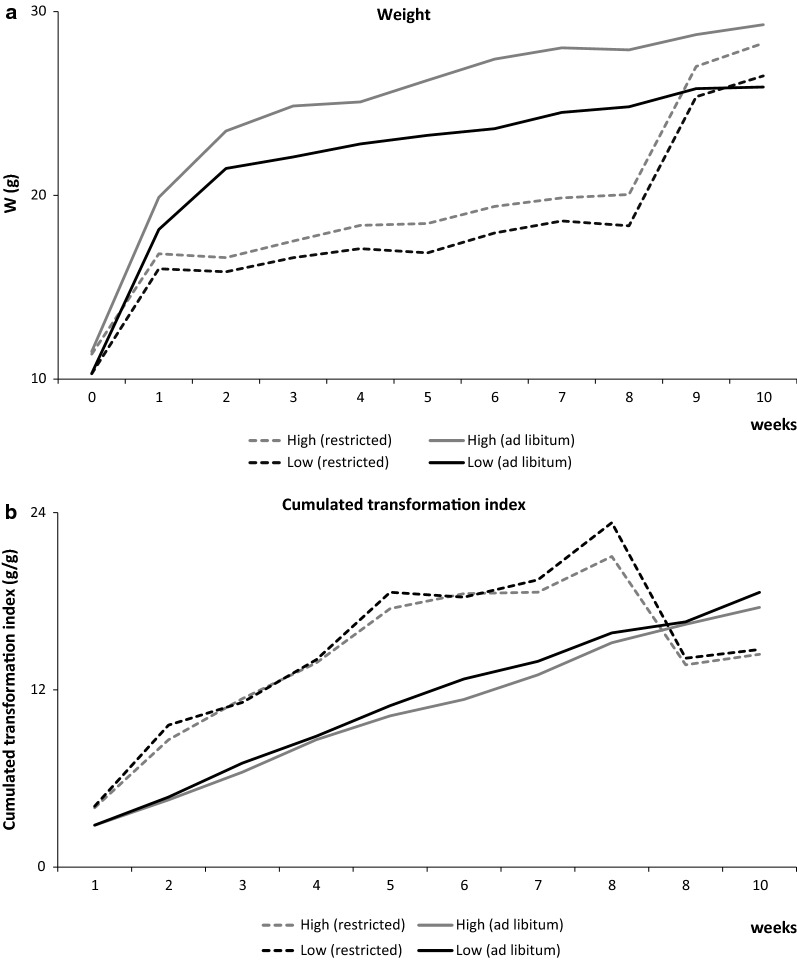
Phenotypic evolution of the female weekly weight (in g) (**a**) and the cumulated transformation index (**b**) depending on feeding regimen (ad libitum or restricted) in both lines with high and low environmental variability of BW from weaning to 10 weeks later

Figure 3a, b show the evolution of female weight (Fig. 3a) and of the cumulated transformation index (Fig. 3b) for each line and feeding regime from 0 to 10 weeks after weaning. The effect of feed restriction was similar in both lines but animals from the low line recovered their normal body weight in one or 2 weeks after being put back on the ad libitum feeding regimen, whereas those from the high line continued to have a slightly smaller body weight than individuals from the same line that were permanently fed ad libitum (Fig. 3a). The cumulated transformation index was similar for both lines regardless of the feeding regimen. After a short recovery period, females that had been subjected to feed restriction reached a lower (better) transformation index value than females that were never feed-restricted, in both lines (Fig. 3b). This compensatory growth effect could be interesting for application to livestock.

Table 3 shows the significance levels for the effects of generation, line and feeding regimen in the growth and reproduction periods, as well as their first order interactions, on fertility (%) in both parturitions (P1 or P2). The effect of line was highly significant (*p* < 0.001) for both parturitions. The other effects were more important in P2 than P1, except in generation 12, in which a stronger feed restriction was applied.

**Table 3:** Significance between lines, feeding regimen (ad libitum or restricted) during the growth period (R_GP) and the reproduction period (R_RP), generation (gen) and its first order interactions for the number of females having one (P1) and two (P2) parturitions

	P1				P2			
	Total	Generation			Total	Generation		
		12_(75%)_	13_(90%)_	14_(85%)_		12_(75%)_	13_(90%)_	14_(85%)_
Line	***	**	**	ns	***	***	**	*
R_GP	*	**	ns	ns	ns	ns	*	ns
R_RP	ns	***	ns	ns	***	***	***	*
Line*R_GP	ns	ns	ns	ns	ns	ns	ns	ns
Line*R_RP	ns	ns	ns	ns	***	**	***	ns
R_GP*R_RP	ns	ns	ns	ns	**	ns	**	ns
Gen	*				*			
Gen*line	ns				ns			
Gen*R_GP	*				ns			
Gen*R_RP	***				**			

*ns* not significant

*p < 0.05; **p < 0.01; ***p < 0.001

The raw data for overall fertility (%) for each combination of line, generation, and feeding regimen during reproductive and growing periods are in Table 4 for P1 and P2. The statistical test refers to differences in feeding regimen. Fertility of animals from the low line was higher than that of animals from the high line. Concerning feeding regimen, feed-restricted females performed worse than those fed ad libitum, except at P1 for high line females of generation 14 that were feed-restricted during the reproduction period. Table 4 also shows that the negative influence of feeding restriction on fertility was stronger when it was applied during the reproduction period than during the growth period, thus reducing the percentage of animals that had a P2. When the restriction was applied during the reproduction period, the number of P2 differed significantly between lines. Feed restriction seemed to affect animals from the high line more, regardless of the physiological stage, growth or reproduction, but these differences were not significant.

**Table 4:** Fertility rate (%) according to feeding regimen (ad libitum or feed restriction) during the growth period (R_GP) and the reproduction period (R_RP) in mouse lines with high and low environmental variability of BW

Period	Generation	Feeding regimen	High line				Low line			
			First birth	*p*	Second birth	*p*	First birth	*p*	Second birth	*p*
R_GP	12_(75%)_	Ad libitum	70	**	10	ns	90	ns	45	ns
		Restriction	35		10		65		30	
	13_(90%)_	Ad libitum	80	ns	25	ns	95	ns	45	ns
		Restriction	55		10		95		30	
	14_(85%)_	Ad libitum	65	ns	0	ns	85	ns	20	ns
		Restriction	80		10		80		25	
R_RP	12_(75%)_	Ad libitum	80	***	20	*	90	ns	70	***
		Restriction	25		0		65		5	
	13_(90%)_	Ad libitum	60	ns	30	*	100	ns	75	***
		Restriction	75		5		90		0	
	14_(85%)_	Ad libitum	65	ns	10	ns	80	ns	35	ns
		Restriction	80		0		85		10	

*ns* not significant

*p < 0.05; **p < 0.01; ***p < 0.001

### Birth weight of offspring

Table 5 shows the significance of the systematic effects and the estimated variances of the random effects for the mean and the variability of BW. The genetic correlation between the location and dispersion parameters for BW was 0.34, but the standard error of this estimate was very large due to the numerous factors of variation and to the limited amount of data.

**Table 5:** Significance of the systematic effects and estimated variances of the random effects affecting both the mean BW (*µ*) and the environmental variability (*v*) and its genetic correlations (ρ)

	BW (*µ*)	BW (*v*)	ρ
Sex	***	***	
LS_pup_	***		
PN	*		
Gen*R_RP	***		
R_GP*R_RP	ns		
Line*R_RP		ns	
$\sigma_{l}^{2}$	0.0198 (0.0028)		
$\sigma_{m}^{2}$	0.0061 (0.0029)	0.0454 (0.0163)	0.34 (0.33)

*LS*_*pup*_ litter size, *PN* parturition number, *gen* generation, *R_GP* growth period, *R_RP* reproduction period, $\sigma_{l}^{2}$ litter variance, $\sigma_{m}^{2}$ maternal genetic variance, *ns* no significant

*p < 0.05; ***p < 0.001

**Fig. 4:**
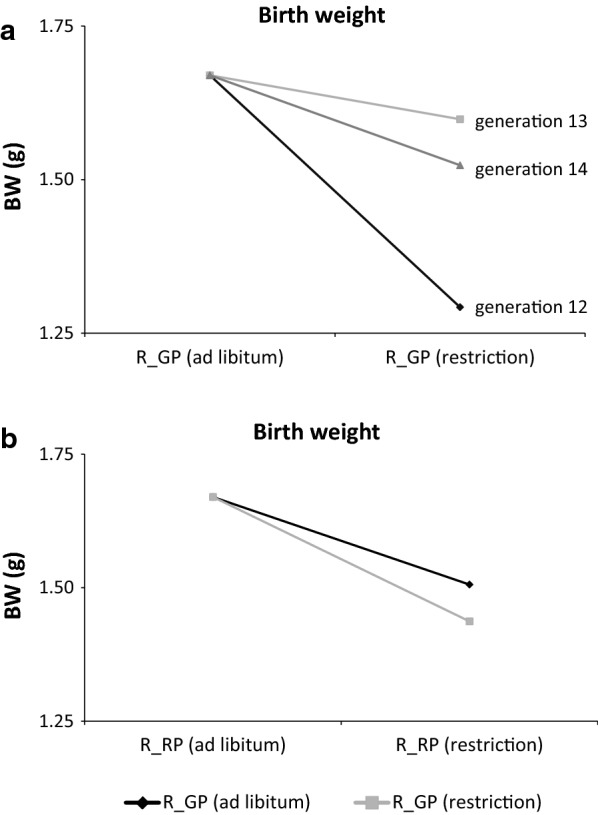
Birth weight of pups (BW) depending on the feeding regimen (ad libitum or restricted) of the dam during the growth period (R_GP) (**a**) and the reproduction period (R_RP) (**b**) in the three generations

Regarding the effect of feed restriction in dams on the BW of their offspring, the effect of offspring generation was significant only for the offspring of mothers that were feed-restricted during the growth period. For these feed-restricted dams, the BW of their pups decreased as the level of feed restriction of the dams increased (Fig. 4a). Similarly, as shown in Fig. 4b, the effect of feed restriction of the mothers during their growth did not affect the BW of their pups, when they were fed ad libitum during the reproduction period. Nevertheless, when the females were feed-restricted during the reproduction period, pups from the females that were fed ad libitum during their growth had a slightly higher BW, than those had been feed-restricted during the same period.

**Fig. 5:**
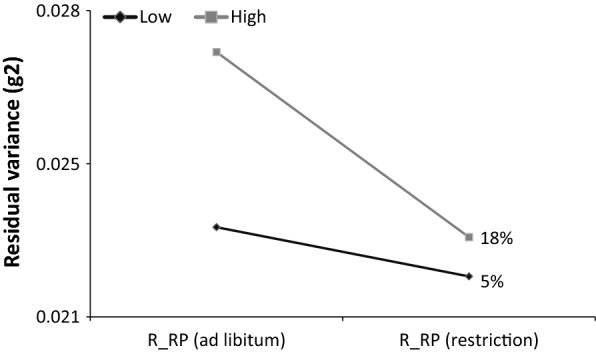
Drop in residual variance ($\sigma_{{e_{sl} }}^{2}$) in the lines with low and high environmental variability of BW depending on feeding regimen (ad libitum or restricted) during the reproduction period (R_GP)

Among the effects that influence the variability of BW, only sex and the interaction between line and feeding regime during the reproduction period were significant (Table 5). This interaction led to different residual variances between the combinations of line and feeding regimens during reproduction, as shown in Table 6, with corresponding estimates of residual variances in Fig. 5. Note that from the ad libitum to the restricted feeding regimen, the residual variance decreased more in the high line (18%) than in the low line (5%).

**Table 6:** Solutions of the interaction between line and feeding regimen (ad libitum or restricted) during the reproduction period (R_RP) effect for the environmental variability ($\eta_{line*R\_RP}$) and corresponding residual variance ($\sigma^{2}$)

Line	Feeding regimen	$\eta_{line*R\_RP}$	$\sigma^{2} = e^{\eta }$
High	Ad libitum	− 3.61	0.027
	Restricted	− 3.78	0.023
Low	Ad libitum	− 3.77	0.025
	Restricted	− 3.82	0.022

## Discussion

We hypothesized that the effect of a feed restriction challenge would be stronger in the line selected for a high than for a low environmental variability of BW, which could be interpreted as the low line being more robust. Although our analysis was on mice that were divergently selected for variability of BW, this could reflect the advantages of selection for decreased variability of a particular trait on robustness and animal welfare in livestock production. Although there is no agreement in the definition of robustness, Knap [25] defines it as the capacity of achieving a high level of productive potential and supporting, at the same time, a certain level of stress, or expressing high productive level in different environments. Mormede and Terenina [2] expressed the same idea in a different way by suggesting that it is a combination of a high production potential with a low sensitivity towards environmental changes.

Formoso-Rafferty et al. [1] showed that animals from the low line were more robust and expressed higher animal welfare and reproductive performance. They mentioned the need to study whether homogeneity could also be desirable under environmental challenges. Thus, the aim of our research was to study the relationship between homogeneity and robustness in a stressful environment. We tried to achieve this by assessing the relationships that exists between a very important production feature, i.e. feed efficiency and homogeneity and robustness. Silalahi et al. [26] argued that animals selected for high production efficiency might be more susceptible to behavioral, physiological, or immunological problems, thus making them less robust [27–29].

In the present experiment, the level of feed restriction had to be modified at each generation, because of unexpected and undesired observations in the initial generations. Thus, we applied a feeding regimen that consisted of 75, 90, and 85% of ad libitum intake in the first, second, and third generations of the experiment, respectively. As a result, the effects of feed-restriction were expected to differ between generations, with a stronger effect in the first generation. However, even in these last two generations, the feed-restrictions applied were expected to generate the desired challenged environment. Although in the design used here, the effect of feed-restriction level could be influenced partly by other causes associated with generation and selection effects, we assumed that the observed differences between generations were mainly due to feed-restriction levels.

### Growth and fertility

The results show that, although the two lines did not statistically differ in weight after weaning, there was a significant interaction between line and feeding regime during the growth period (Table 2). This interaction, which was observed from week 2 after weaning, could be interpreted as a consequence of a cumulated negative effect from the previous weeks. Figure 2 shows the least square means of the weights in week 4 after weaning, at which time animals had reached maturity (i.e. at 49 days of age [30]). Although there were differences in body weight between the lines, they were not significant (Table 2); animals from the low line were lighter than those from the high line within feeding regimen and generation, as shown in Fig. 2. The smaller weight of animals from the low line under ad libitum feeding had already been reported based on a larger number of animals [1, 31]. The differences between animals fed ad libitum and feed-restricted were always higher for the high than for the low line. Thus, our results suggest that animals from the low line are less sensitive to a change in feed level than those from the high line.

In some cases, a low level of feed-restriction can have a beneficial effect. Young mammals [18] are prone to digestive diseases, particularly around the weaning period. Feeding practices such as restricted feeding are known to affect physiological and productive traits in growing rabbits [18, 19, 32]. Lu et al. [33] reported that, after returning to ad libitum feeding, feed-restricted animals can show compensatory growth [34, 35], increased nutrient digestibility [16, 36], and increased feed conversion rate [37]. This compensatory growth depends on the duration, level, and pattern of feed restriction [16, 17, 36, 38]. The effect of feed-restriction on growth was more easily compensated for, when the duration of feed restriction was short or moderate. We observed that the high line was more negatively affected by a greater level of feed restriction than the low line. Although feed restriction affected the growth of both lines similarly, animals from the low line recovered their normal weight in one or 2 weeks, whereas those from the high line did not attain a normal weight at the end of the experiment (Fig. 3a). Nevertheless, it is generally assumed that a very low level of feed restriction could prevent animals from fattening, and this effect seemed to be more beneficial for the high than the low line since feed restriction increased reproductive performance in the high line and not to the same extent in the low line.

### Birth weight of offspring

The effect of feed restriction during the reproduction period was greater for females that had also been feed-restricted during their growth period, thus manifesting a memory effect. However, we would not have observed this differential effect of feed restriction during the reproduction period, if the experiment had been designed to feed-restrict animals only during the growth period. In spite of the recovery capacity of females, the low effect on the BW of their own pups made the memory effect patent, confirming previously reported results in rats [39] and cattle [40].

The estimate of the variance of the litter effect was threefold greater than the genetic variance of the maternal effect, which was small but different from zero. This result is in agreement with that previously reported during the selection process [14].

Overall, selection for decreased environmental variability of BW appeared to have conferred a lower sensitivity of the mothers to the changing environmental conditions. Animals from the high line were more sensitive to harsh feeding restrictions than those from the low line. However, a moderate feeding restriction prevented the high line females from fattening, resulting in fertility rates that were comparable between lines, as shown in generation 14 (Table 4).

The estimate of the genetic correlation between the mean and dispersion parameters for BW was 0.34 (Table 5), which is similar to that obtained by Formoso-Rafferty et al. [7] on the same population but with a higher accuracy. Because of this correlation, the mean BW of the offspring was greater for the high line than for the low line, as shown by Formoso-Rafferty et al. [7].

The genetic component of the environmental variance can be related to the capacity of animals to adapt to new environmental conditions, which can affect their welfare [41]. There is some evidence that environmental variability is under genetic control [7]. In addition to the parameters estimated in several datasets [7], two selection experiments that used variability of BW as a selection criterion were performed with success, one in mice [14] and one in rabbits [13].

Feed costs represent the largest financial input in livestock production systems but can be reduced by reducing feed intake [42, 43]. However, limiting feed intake is also expected to reduce production and, therefore, livestock must be selected based on feed efficiency rather than only on feed intake [44]. Based on our findings, animals that show less environmental variability of BW are more robust, which means that they cope better with the environmental challenge of feed restriction.

Blasco et al. [43] demonstrated that a rabbit line selected for homogeneity in litter size tolerated external stressors more effectively than the line selected for heterogeneity in litter size. Thus, in general, animals selected for increased environmental variability appear to be more sensitive to stress, have a lower immune response to infections, and a higher hepatic activity [45]. These effects have consequences on disease resistance but also on animal welfare, because animals that cope more effectively with their environment have better welfare than animals that are more sensitive. Using the definition by Mormede and Terenina [2] and the conclusions in Formoso-Rafferty et al. [1], our findings demonstrate a relationship between low environmental variability and robustness in a challenging environment. We also show that the low line has a higher survival rate and better fertility even when the animals were feed-restricted. Finally, the low line is more stable and better able to tolerate stressful conditions.

The mouse is frequently used as a model animal in selection experiments because of its favorable characteristics such as short generation intervals and large number of pups per parturition. Conclusions based on mice can be extrapolated to livestock species such as pigs or rabbits [46]. A research project on homogeneity in sheep is in progress in which the selection index has been modified to include a specific weight for this selection objective [47]. More research is needed to ensure the suitability of applying this type of selection in cattle.

## Conclusions

When an environmental challenge in the form of a feed restriction was applied, the mouse line selected for a low environmental variance of BW had a higher survival rate and better reproductive efficiency than the high line. The animals from the low line were less affected by the feed restriction and were more robust to this environmental challenge than those from the high line.

## Data Availability

The datasets generated and/or analyzed during the current study are available from the corresponding author upon reasonable request.
